# Nebivolol attenuates the anticonvulsant action of carbamazepine and phenobarbital against the maximal electroshock-induced seizures in mice

**DOI:** 10.1007/s43440-019-00029-6

**Published:** 2019-12-20

**Authors:** Kinga K. Borowicz-Reutt, Monika Banach, Monika Rudkowska

**Affiliations:** Independent Unit of Experimental Neuropathophysiology, Department of Pathophysiology, Medical University of Lublin, Jaczewskiego 8, PL-20-954 Lublin, Poland

**Keywords:** Nebivolol, Classical antiepileptic drugs, Drug interactions, Electroshock maximal

## Abstract

**Background:**

Due to co-occurrence of seizures and cardiovascular disorders, nebivolol, a widely used selective β_1_-blocker with vasodilatory properties, may be co-administered with antiepileptic drugs. Therefore, we wanted to assess interactions between nebivolol and four conventional antiepileptic drugs: carbamazepine, valproate, phenytoin and phenobarbital in the screening model of tonic–clonic convulsions.

**Methods:**

Seizure experiments were conducted in the electroconvulsive threshold and maximal electroshock tests in mice. The chimney test served as a method of assessing motor coordination, whereas long-term memory was evaluated in the computerized step-through passive-avoidance task. To exclude or confirm pharmacokinetic interactions, we measured brain concentrations of antiepileptic drugs using the fluorescence polarization immunoassay.

**Results:**

It was shown that nebivolol applied at doses 0.5–15 mg/kg did not raise the threshold for electroconvulsions. However, nebivolol at the dose of 15 mg/kg reduced the anti-electroshock properties of carbamazepine. The effect of valproate, phenytoin, and phenobarbital remained unchanged by combination with the β-blocker. Nebivolol significantly decreased the brain concentration of valproate, but did not affect concentrations of remaining antiepileptic drugs. Therefore, contribution of pharmacokinetic interactions to the final effect of the nebivolol/carbamazepine combination seems not probable. Nebivolol alone and in combinations with antiepileptic drugs did not impair motor performance in mice. Nebivolol alone did not affect long-term memory of animals, and did not potentiate memory impairment induced by valproate and carbamazepine.

**Conclusions:**

This study indicates that nebivolol attenuated effectiveness of some antiepileptic drugs. In case the results are confirmed in clinical settings, this β-blocker should be used with caution in epileptic patients.

## Introduction

Based on many studies, a bidirectional relationship between cardiac arrhythmias and seizures was established. On the one hand, cardiac arrhythmias are well-known causes of acute seizure-like activity. In very serious cases, for example asystole, true seizures can be triggered. On the other hand, so-called autonomic seizures are frequently accompanied by cardiac arrhythmias. The mechanism of this phenomenon is based on interactions between seizures and the function of the autonomic nervous system. During a seizure, abnormal burst of electrical activity spread in the brain can affect centers for the regulation of autonomic activity, including the insula, amygdala, cingulated gyrus, and prefrontal cortex. Depending on the location of the epileptic focus, the sympathetic or parasympathetic system may be modulated more. Such an autonomic dysregulation is usually manifested not only by vagal suppression (often leading to cardiorespiratory dysfunction), but also sympathetic activation, vagal activation, and sympathetic–vagal suppression [1]. The most frequently described cardiorespiratory symptoms are: tachyarrhythmia with LQTS (long QT syndrome) and bradycardia with dyspnea/apnea syndrome [2, 3]. Moreover, it was reported that status epilepticus (SE) can lead to an increase in blood pressure and/or cardiac failure [4]. In general, autonomic cardiorespiratory disorders are believed to play a crucial role in the pathogenesis of sudden unexpected death in epilepsy (SUDEP) [5, 6]. On the other hand, uncontrolled hypertension, probably in the mechanism of vascular brain damage, may increase the risk of new-onset seizures even in patients without any symptoms of forthcoming stroke [7].

The contribution of noradrenergic neurotransmission to epileptogenesis and seizure manifestation seems indisputable. In in vitro conditions, central activation of β-adrenoceptors may facilitate excitatory amino acids release and trigger seizures [8]. Hippocampus, strongly involved in seizure generation and propagation, has the highest density of β_1_ and β_2_ receptors among other brain structures [9]. Several β-blockers, primarily propranolol, metoprolol, pindolol, timolol and nebivolol, were reported to inhibit experimental seizures. Propranolol, metoprolol and nebivolol potentiated the anticonvulsant action of antiepileptic drugs in electrical and sound-induced seizure tests in mice [1]. Because of the potentially beneficial action of β-adrenoceptor antagonists in seizures and relationship between seizures and arrhythmias, antiepileptic drugs and β-blockers may be co-administered in patients suffering of both disorders. It is worth mentioning that atenolol, another β-receptor antagonist, has prevented dysfunctions of cardiovascular system observed in rats with status epilepticus [4].

Nebivolol is a potent, cardioselective and long-acting III generation β-blocker with unique vasodilatory, antioxidative and antiplatelet actions [10]. Antioxidative action and high lipophilicity, and good permeability through the blood–brain barrier contribute to central effects of nebivolol [11, 12]. Actually, nebivolol and other lipophilic β-blockers are frequently used for the treatment of migraine and essential tremor [12]. Such properties also make the effect of nebivolol on seizures very probable. At present, usage growth rate for nebivolol is the highest among all β-blockers. This medication is recommended particularly in the treatment of hypertension, ischemic heart disease and heart failure. Contrary to many other β-blockers (e.g., atenolol), nebivolol mostly decreased the level of serum triglycerides, exhibited a favorable effect on the cholesterol profile and improved glucose metabolism in clinical conditions. Moreover, insulin resistance is commonly related to endothelial dysfunction and reduced nitric oxide availability. Therefore, nebivolol is also predisposed to be used in diabetic patients [10].

Among available data, there is only one report describing the action of nebivolol on experimental convulsions. According to authors, this β-blocker elevated the seizure threshold and potentiated the action of lamotrigine in the model of increasing current electroshock seizures (ICES) in mice [13]. This encouraged us to evaluate the effect of nebivolol on the anticonvulsant action of conventional antiepileptic drugs in the maximal electroshock test, the leading animal model of tonic–clonic seizures and screening test for potential anticonvulsant agents.

## Materials and methods

### Animals

Female Swiss mice weighing 20–25 g were used in this study. Animals were kept in standard laboratory conditions with a natural dark–light cycle and housed in colony cages providing free access to tap water and food (Experimental Medicine Center, Medical University of Lublin). Experimental procedures were conducted between 9 a.m. and 2 p.m. Control and experimental groups consisted of 8 animals. All experiments designed in this study were compliant with EU Directive 2010/63/EU for animal experiments and approved by the Local Ethical Committee for the Animal Experiments at the University of Life Science in Lublin (Licence Number: 42/2015).

### Drugs

In the present study, we used nebivolol (NEB), an antiarrhythmic medication, and four antiepileptic drugs: valproate (VPA), carbamazepine (CBZ), phenytoin (PHT) and phenobarbital (PB). VPA, CBZ and PHT were obtained from Sigma (St. Louis, MO, USA), while PB from UNIA Pharmaceutical Department (Warsaw, Poland). Among all antiepileptic drugs, only VPA was dissolved in sterile saline; whereas, CBZ, PHT, PB and NEB were suspended in a 1% solution of Tween 80 (Sigma, St. Louis, MO, USA) in saline. All drugs used in the study were applied intraperitoneally (*ip*) in a single injection and in a volume of 10 ml/kg. Injections were made in a specified time before the behavioral tests: PHT—120 min, PB—60 min, VPA, CBZ, and NEB—30 min before the tests. Time between drug application and experimental procedures was established experimentally, as time of the maximum effect against electroconvulsions (e.g., [14]).

### Electroconvulsive threshold and maximal electroshock seizure test in mice

Maximal electroshock test (MES) is the most frequently used standard preclinical model of tonic–clonic seizures [15]. Details of MES-related procedures were extensively described by authors from our lab, for instance Borowicz et al. [14]. In brief, to evaluate the threshold for maximal electroconvulsions, at least 4 groups of mice, consisting of 8 animals per group, were challenged with electroshocks of various current intensities ranging between 5 and 9 mA, to yield 10–30%, 30–50%, 50–70%, and 70–90% of animals with seizures. Then, a current intensity vs. response curve was constructed, according to a log-probit method by Litchfield and Wilcoxon [16], from which a median current strength (CS_50_ in mA) was calculated. Each CS_50_ value represents the current intensity required to induce tonic hind limb extension in 50% of the mice challenged.

The anti-electroshock properties of four classical antiepileptic drugs and their combinations with nebivolol were expressed as their ED_50_s (median effective doses against MES). Electrical current with strictly defined parameters (stimulus with current intensity of 25 mA lasting for 0.2 s) was delivered to mice by ear-clip electrodes. The animals were administered different antiepileptic drug doses so as to obtain a variable percentage of protection against seizures, allowing for the construction of a dose–response curve. The respective ED_50_ values (mg/kg) were calculated based on the formula of Litchfield and Wilcoxon [16].

### Chimney test

This test was employed to define effects of nebivolol alone and in combinations with classical antiepileptic drugs on motor coordination in mice [17]. Detailed test methodology was accurately described in our previous studies [14]. Mice subjected to the test were administered with antiepileptic drugs alone, nebivolol alone or combinations of nebivolol with respective antiepileptic. Antiepileptic drugs were applied at their ED_50_s, while nebivolol at its highest dose used in this study (15 mg/kg).

### Step-through passive avoidance task

This test measuring long-term memory is based on natural in rodents avoidance of bright rooms. Detailed description of the whole procedure was provided previously [14]. In this study, however, we used for the first time a fully automated apparatus with specific hardware and software features (Multi Conditioning System, TSE Systems GmbH, Bad-Homburg, Germany). The MCS software features are compliant with the Good Laboratory Practice. This apparatus allows entire isolation of animals from external stimuli that may interfere with mouse behavior. Therefore, test results are more reliable when compared to manual method. Animal’s behavior is continuously observed on the monitor through a camera placed inside the chamber. A punishing electrical stimulus (0.3 mA for 2 s) was triggered in the dark compartment by rods in a greed floor.

Like in the chimney test, antiepileptic drugs were applied alone at their ED_50_s or in combinations with nebivolol (15 mg/kg). Results were presented as medians (with 25, 75 percentiles) of time needed by animals to enter the dark box.

### Measurement of brain concentrations of antiepileptic drugs

Fluorescence polarization immunoassay was used to measure brain concentrations of classical antiepileptic drugs used in this study. Control animals were injected with a combination of one of the antiepileptic drugs and saline. Experimental groups received nebivolol (15 mg/kg) instead of saline. Subsequently, the mice were decapitated at times set for the MES test. Brains were isolated and homogenized by Ultra Turax T8 homogenizer (IKA, Staufen, Germany) with Abbott buffer (2:1 vol/weight). Finally, after brain homogenate centrifugation (10,000 g for 15 min), the content of drugs in supernatants (75 μl) was measured by Architect c4000 clinical chemistry analyzer (Abbott Laboratories Poland). Concentrations of antiepileptic drugs were presented as mean ± SD of at least eight determinations and expressed in μg/ml.

### Statistics

In the MES test, ED_50_ values with 95% confidence limits were calculated based on computer log-probit analysis of Litchfield and Wilcoxon [16]. Then, confident limits were converted to standard errors (SEMs) and respective ED_50_ values of control and experimental groups were compared with the one-way ANOVA with post hoc Sidak multiple comparison test.

Qualitative variables obtained from the chimney test were compared statistically using Fisher’s exact probability test. Results from the computerized step-through passive-avoidance task were analyzed in the Kruskal–Wallis nonparametric ANOVA test followed by the post hoc Dunn’s test.

Changes in brain concentrations of respective antiepileptic drugs were evaluated using the unpaired Student’s *t* test. In all tests, the significance level was set at *p *≤ 0.05.

## Results

### Electroconvulsive threshold

Nebivolol, applied at doses ranging from 0.5 to 15 mg/kg, did not change the value of electroconvulsive threshold in mice. However, an insignificant tendency to decrease the threshold was observed. The control value was assessed as 5.1 ± 0.50 mA, while nebivolol (15 mg/kg) reduced it to 4.4 ± 0.23 mA (Table 1). The dose of nebivolol from which we started experiments (0.5 mg/kg) was established on the basis of the study of Goel et al. [13], where this β-blocker (at the dose of 0.5 mg/kg) significantly increased the threshold of increasing current electroshock seizures (ICES) in mice. ICES, similarly to the maximal electroshock seizure test, is a screening rodent model of tonic–clonic convulsions [18]. We decided not to exceed the 15 mg/kg dose. According to mathematical translation [19], larger doses would be much higher than therapeutic doses recommended in patients.

**Table 1:** Effects of nebivolol on the electroconvulsive threshold in mice

Treatment (mg/kg)	CS_50_ (mA)	Statistics (ANOVA)
Vehicle	5.1 ± 0.36	*F* (5.104) = 1435; *p* = 0.218
NEB (0.5)	5.2 ± 0.33	
NEB (2,0)	5.2 ± 0.41	
NEB (5,0)	5.4 ± 0.18	
NEB (10.0)	4.5 ± 0.14	
NEB (15.0)	4.4 ± 0.23	

Results are expressed as values of current strength that induced convulsions in 50% of mice (CS_50_) ± SEM. NEB, nebivolol

### Maximal electroshock test

In the MES test, nebivolol was combined with classical antiepileptic drugs. To limit the number of animals used in the study, according to guidelines of the Ethical Committee, we started from the dose of 5 mg/kg.

**Fig. 1:**
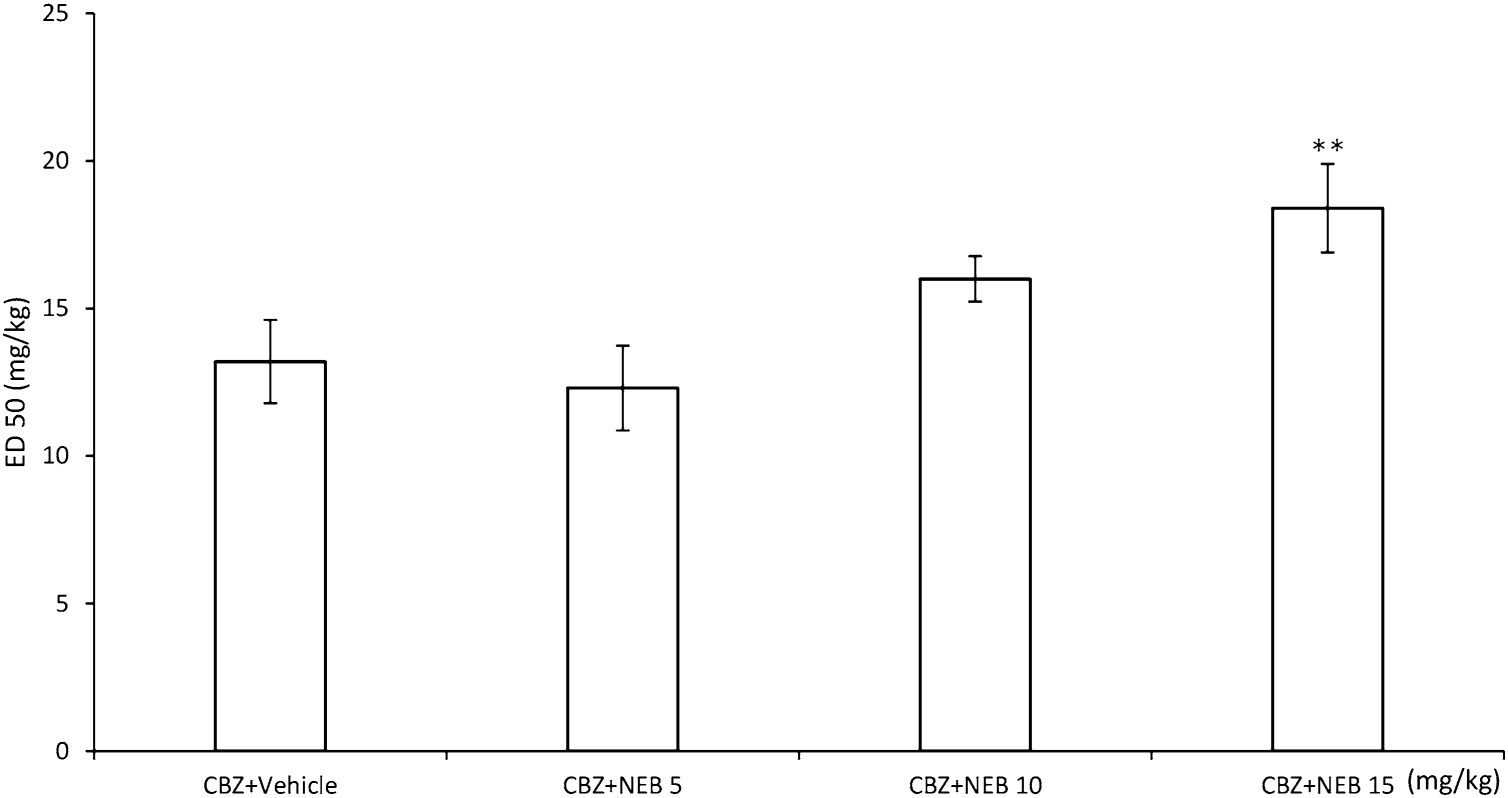
Effect of nebivolol (NEB) on the anticonvulsant action of carbamazepine (CBZ) against maximal electroshock-induced seizures in mice. Data are presented as median effective doses (ED_50_s with SEM values), at which CBZ alone and in combinations with NEB protected 50% of animals against seizures. **F (3.128) = 4.02; *p* = 0.009 versus control (animals treated with CBZ plus vehicle)

Nebivolol applied at doses of 5 and 10 mg/kg did not affect the anti-electroshock activity of classical antiepileptic drugs. The β-blocker at the dose of 15 mg/kg did not influence the action of valproate, phenytoin, and phenobarbital (Table 2). At the same dose, however, it reduced the anticonvulsant properties of carbamazepine: *F* (3.128) = 4.02; *p* = 0.009 (Fig. 1). Interestingly, nebivolol had also a tendency to increase the ED_50_ dose of phenobarbital and phenytoin; however, this effect occurred insignificant.

**Table 2:** Effects of nebivolol on the anti-electroshock action of valproate and phenytoin

Treatment (mg/kg)	ED_50_ (mg/kg)	Statistics (ANOVA)
VPA + vehicle	356.9 ± 14.92	*F* (3.104) = 1.275; *p* = 0.2871
VPA + NEB (5)	320.2 ± 10.46	
VPA + NEB (10)	348.9 ± 13.71	
VPA + NEB (15)	353.1 ± 10.80	
PHT + vehicle	13.3 ± 1.52	*F* (3.140) = 0.7035; *p* = 0.5514
PHT + NEB (5)	11.3 ± 0.72	
PHT + NEB (10)	14.3 ± 0.84	
PHT + NEB (15)	15.4 ± 0.93	
PB + vehicle	24.1 ± 1.84	*F* (3.112) = 2.109; *p* = 0.1031
PB + NEB (5)	25.5 ± 2.03	
PB + NEB (10)	24.5 ± 1.92	
PB + NEB (15)	31.9 ± 1.23	

Results are expressed as 50% effective doses (ED_50_) ± SEM

*VPA* valproate, *PHT* phenytoin, *PB* phenobarbital, *NEB* nebivolol

### Chimney test and passive-avoidance task

Classical antiepileptic drugs administered alone (at their ED_50_ doses) or in combinations with nebivolol (15 mg/kg) did not influence motor performance of mice tested in the chimney test. The greatest motor impairment (observed in 30% of mice) did not reach the level of significance (*p* = 0.2105).

Nebivolol, phenytoin and phenobarbital administered alone did not impair long-term memory in mice. In contrast, carbamazepine alone (18.4 mg/kg) or in combination with nebivolol (15 mg/kg) significantly weakened cognitive functions: *H*(2) = 10.079, *p* = 0.0065. Memory deficits were also essential in the group treated with valproate (353 mg/kg) alone or in combination with nebivolol (15 mg/kg): *H*(2) = 14,215, *p* = 0.0008. Similarly, the combination of phenytoin (15.4 mg/kg) with nebivolol (15 mg/kg) significantly decreased long-term memory: *H*(2) = 6.267, *p* = 0.0436. Relevant but insignificant memory changes were observed in the case of the combined treatment of phenobarbital (31.9 mg/kg) with nebivolol (15 mg/kg): *H*(2) = 5.346, *p* = 0.0691. Nevertheless, in no case, nebivolol potentiated memory impairment induced by antiepileptic drugs (Table 3).

**Table 3:** Effects of conventional antiepileptic drugs administered alone or in combinations with nebivolol on long-term memory and motor coordination

Treatment (mg/kg)	Median (25, 75 percentiles)	Mice impaired (%)
Vehicle	180 (178.6, 180)	0
NEB (15)	47.2 (34.8, 118.2)	0
VPA (353) − ED_50_	29.4 (17.6, 100)**	10
VPA (353) + NEB (15)	31 (19, 55)**	30
PHT (15.4) − ED_50_	44.4 (11.4, 180)	0
PHT (15.4) + NEB (15)	27.6 (13.8, 69.8)*	10
CBZ (18.4) − ED_50_	46.5 (14.7, 65.2)*	0
CBZ (18.4) + NEB (15)	35.2 (20.2, 91.9)*	0
PB (31.9)	26.7 (19.9, 180)	10
PB (31.9) + NEB (15)	46 (24.9, 154.5)	10

Results are shown as percentage of animals showing motor deficits in the chimney test and as median retention times (with 25th and 75th percentiles in parentheses) observed in the step-through passive-avoidance task. Statistical analysis of data from the chimney test was performed with Fisher’s exact probability test, whilst data from the passive-avoidance test were evaluated by use of the Kruskal–Wallis nonparametric ANOVA test followed by the post hoc Dunn’s test. NEB, nebivolol; VPA, valproate; PHT, phenytoin; CBZ, carbamazepine; PB, phenobarbital

*p < 0.05, **p < 0.01 versus control (vehicle-treated mice). For detailed statistical data see chapter 3.3

### Plasma and brain concentrations of antiepileptic drugs

Nebivolol (15 mg/kg) decreased the brain concentration of valproate. No significant changes were observed in the case of carbamazepine, phenytoin or phenobarbital (Table 4).

**Table 4:** Brain concentrations of conventional antiepileptic drugs applied alone and in combinations with nebivolol

Treatment (mg/kg)	Brain concentration (μg/ml)	Statistics
VPA (353) + vehicle	88.31 ± 8.24	NA
VPA (353) + NEB (15)	78.02 ± 8.70*	*t* (18) = 2.716; *p* = 0.0142
PHT (15.4) + vehicle	0.65 ± 0.20	
PHT (15.4) + NEB (15)	0.82 ± 0.25	*t* (16) = 1.593; *p* = 0.1307
CBZ (18.4) + vehicle	2.04 ± 0.80	
CBZ (18.4) + NEB (15)	1.41 ± 0.53	*t* (18) = 2.076; *p* = 0.0525
PB (31.2) + vehicle	5.03 ± 0.64	
PB (31.2) + NEB (15)	5.30 ± 1.14	*t* (16) = 0.6196; *p* = 0.5443

Data are presented as mean ± SD of at least eight determinations. Statistical analysis of the brain concentrations of antiepileptic drugs was performed using the unpaired Student’s *t* test

*NEB* nebivolol, *CBZ* carbamazepine, *VPA* valproate, *PHT* phenytoin, *PB* phenobarbital, *NA* not applicable

*p < 0.05 versus control (the respective antiepileptic drug)

## Discussion

Results presented herein show that nebivolol administered per se in doses up to 15 mg/kg did not affect the electroshock seizure threshold. However, at the highest dose, it decreased the anti-electroshock action of carbamazepine. This effect is probably pharmacodynamic by nature, since nebivolol did not change brain concentrations of the two antiepileptic drugs.

Results of our study seem to be rather unexpected. According to Goel et al. [13], nebivolol at the much lower dose of 0.5 mg/kg increased the electrical threshold and potentiated the action of lamotrigine in the ICES test. Therefore, we looked forward to similar effect, especially that both MES and ICES are recognized as models of generalized tonic–clonic convulsions. Possible cause of such discrepancies may lie, for instance, in different current parameters. In the MES, we used 50-Hz current, whereas Goel et al. [13] did not specify the frequency value. Nevertheless, the ICES threshold was reported to be of 12.8 mA. In our study, the control value of the electroconvulsive threshold was much lower (of 5.1 mA). Therefore, results obtained even in very similar seizure tests are not always as convergent as we would expect.

In contrast to nebivolol, some other β-blockers proved significant anticonvulsant action in a variety of seizure models. The most effective in this aspect occurred propranolol, which inhibited seizures induced in mice by: the maximal electroshock [20–22], pentetrazole [23], lidocaine [24], focal penicillin [22], strychnine [25], picrotoxin [26], isoniazid [27], and sound [28, 29]. Such unique properties were mainly attributed to local anesthetic effects of propranolol and its good permeability through the blood–brain barrier. Similarly to propranolol, metoprolol and acebutolol attenuated maximal electroshock- [21] and sound-induced convulsions [29] in mice. Also timolol, used mainly in ophthalmology, inhibited *icv* pentetrazole-induced seizures in cats [30].

Experimental data obtained in both electrically and chemically induced convulsions indicate that β-blockers, regardless of their action on the seizure threshold, may increase effects of antiepileptic drugs [21, 31, 32] (Table 5).

**Table 5:** Summary of data on the action of various β-blockers on the anticonvulsant effect of antiepileptic drugs

β-blocker	VPA	DZP	PHT	GBP	References
Propranolol	+ (MES)	+ (MES)	0	nt	[31]
Metoprolol	+ (MES)	+ (MES)	0	nt	[31]
Sotalol	+ (MES)	nt	+ (MES)	nt	[31]
Acebutolol	+ (MES)	0	0	nt	[21]
Atenolol	nt	+ (AMI)	nt	nt	[33]
Carvedilol				+ (ICES)	[32]
				+ (PTZ)	[32]

*VPA* valproate, *DZP* diazepam, *PHT* phenytoin, *GBP* gabapentin, *MES* maximal electroshock in mice, *ICES* increasing current electroshock seizures in mice, *PTZ* pentetrazole-induced seizures in mice, + intensification of the anticonvulsant effect, 0 no effect on the anticonvulsant effect, *nt* not tested

In clinical settings, the antiseizure effect of propranolol was confirmed in patients with drug-resistant chronically unstable generalized epilepsy [34] or with startle-induced epileptic seizures [35].

Furthermore, β-blockers potentiated also the antiseizure properties of other than antiepileptic medications. For instance, pindolol enhanced the action of fluoxetine in electrically evoked focal hippocampal seizures in rats [36]; propranolol increased the effect of nifedipine in the MES test in mice [37]; while propranolol, metoprolol and atenolol intensified the action of glutamatergic receptor antagonists (MK-801 and/or GYKI 52466) [38].

Mechanism of anticonvulsant action of β-blockers has been tried to be explained by reduced formation of cAMP. Indeed, elevated brain concentrations of cAMP may contribute to seizure generation. Activation of cAMP-dependent protein kinase was reported to enhance both NMDA- and AMPA/kainate-mediated postsynaptic mechanisms of excitotoxicity [38]. Nebivolol exerts an additional antioxidative action [39] that should contribute to the assumed antiseizure effects of this β-blocker [40].

The uniqueness of nebivolol is also related to its agonistic interaction with β_3_ adrenoceptors and, in consequence, stimulating endothelial nitric oxide synthase. Nitric oxide-mediated vasodilation is a reason why nebivolol is recommended in the treatment of hypertension. Although β_3_ receptors are the most widespread in the adipose tissue, their presence was also found in the brain, with the highest density in the hippocampus, cortex and striatum. Some β_3_ agonists were reported to be effective in animal models of depression and anxiety [39]. However, there are no available data on the possible relationship between β_3_ brain adrenoceptor activation and seizures.

In this study, long-term memory impairment was assessed for the first time in the fully automated step-through passive avoidance task (Multi Conditioning System, MCS). Previously, we used for years a manually operated equipment. However, results obtained in the two models were not entirely convergent. The most important finding remained the same: the tested substance, in this case nebivolol, did not enhance memory impairment induced by antiepileptic drugs. Nevertheless, in the less sensitive manually made test, antiepileptic drugs at their ED_50_ doses never caused significant impairment of long-term memory in mice [31]. On the contrary, valproate and carbamazepine markedly worsened memory of animals in the MCS test. It seems that entire isolation of mice from external stimuli allows to receive more reliable results. Findings of this study on memory impairment appear very similar to clinical observations.

As it is widely known, epilepsy itself results in cognitive dysfunction, including memory. A number of studies suggest that anticonvulsant drugs further impair cognitive skills. Polytherapy and prenatal exposure to antiepileptic drugs increase the risk of memory impairment [41, 42]. In clinical conditions, phenobarbital and carbamazepine impaired mostly short-term memory and concentration; phenytoin affected attention, problem solving ability, and performance of visuomotor tasks; whereas, valproate had minimal influence on cognition [43]. Moreover, according to Jokeit et al. [44], patients taking carbamazepine, phenobarbital, and phenytoin at higher therapeutic doses were selectively impaired in the retention but not acquisition of new knowledge and skills.

## Conclusions

In contrast to other β-blockers, caution should be recommended prior to nebivolol use in epileptic patients, at least those treated with carbamazepine or phenobarbital. Nevertheless, further animal and clinical studies are needed to draw more exact conclusions.
