# Correction to: Nebivolol attenuates the anticonvulsant action of carbamazepine and phenobarbital against the maximal electroshock-induced seizures in mice

**DOI:** 10.1007/s43440-021-00275-7

**Published:** 2021-05-28

**Authors:** Kinga K. Borowicz-Reutt, Monika Banach, Monika Rudkowska

**Affiliations:** Independent Unit of Experimental Neuropathophysiology, Department of Pathophysiology, Medical University of Lublin, Jaczewskiego 8, 20-954 Lublin, Poland

## Correction to: Pharmacological Reports (2020) 72:80–86 10.1007/s43440-019-00029-6

The article “Nebivolol attenuates the anticonvulsant action of carbamazepine and phenobarbital against the maximal electroshock-induced seizures in mice”, written by Kinga K. Borowicz-Reutt, et al., was originally published electronically on the publisher’s internet portal on 20 December 2019 without open access. With the author’ decision to opt for Open Choice the copyright of the article changed on 23 April 2021 to © Author(s) 2019 and the article is forthwith distributed under a Creative Commons Attribution 4.0 International License, which permits use, sharing, adaptation, distribution and reproduction in any medium or format, as long as you give appropriate credit to the original author(s) and the source, provide a link to the Creative Commons licence, and indicate if changes were made. The images or other third party material in this article are included in the article’s Creative Commons licence, unless indicated otherwise in a credit line to the material. If material is not included in the article’s Creative Commons licence and your intended use is not permitted by statutory regulation or exceeds the permitted use, you will need to obtain permission directly from the copyright holder. To view a copy of this licence, visit http://creativecommons.org/licenses/by/4.0.

The original article has been corrected.

